# *Caulerpa lentillifera* (Sea Grapes) Improves Cardiovascular and Metabolic Health of Rats with Diet-Induced Metabolic Syndrome

**DOI:** 10.3390/metabo10120500

**Published:** 2020-12-07

**Authors:** Ryan du Preez, Marwan E. Majzoub, Torsten Thomas, Sunil K. Panchal, Lindsay Brown

**Affiliations:** 1 Functional Foods Research Group, University of Southern Queensland, Toowoomba, QLD 4350, Australia; r.dupreez@cqu.edu.au (R.d.P.); S.Panchal@westernsydney.edu.au (S.K.P.); 2 Centre for Marine Science and Innovation, University of New South Wales, Sydney, NSW 2052, Australia; m.majzoub@unsw.edu.au (M.E.M.); t.thomas@unsw.edu.au (T.T.); 3 School of Biological, Earth and Environmental Sciences, University of New South Wales, Sydney, NSW 2052, Australia; 4 School of Health and Wellbeing, University of Southern Queensland, Ipswich, QLD 4305, Australia

**Keywords:** *Caulerpa lentillifera*, sea grapes, green seaweed, gut microbiota, metabolic syndrome

## Abstract

*Caulerpa lentillifera* (sea grapes) is widely consumed in South-East Asia as a low-energy food with high contents of vitamins and minerals. This study investigated dried sea grapes containing 16.6% insoluble fibre commercially produced in Vietnam as an intervention. We hypothesised that insoluble fibre is the primary metabolite that will reverse diet-induced metabolic syndrome. Male Wistar rats (*n* = 48) were randomly allocated to four groups in a 16 week protocol. Two groups were fed either corn starch (C) or high-carbohydrate, high-fat (H) diets for the full 16 weeks. The other two groups received C and H diets for eight weeks and then received *C. lentillifera* added to these diets for the final eight weeks (CCL and HCL, respectively). High-carbohydrate, high-fat diet-fed rats developed obesity, hypertension, dyslipidaemia, fatty liver disease and increased left ventricular collagen deposition. *C. lentillifera* supplementation in HCL rats decreased body weight, systolic blood pressure, plasma concentrations of total cholesterol and non-esterified fatty acids, inflammatory cells in heart and liver, and visceral adiposity. The Firmicutes to Bacteroidetes ratio decreased in the gut microbiota of HCL rats. Therefore, *C. lentillifera* attenuated cardiovascular and metabolic symptoms of metabolic syndrome in rats, possibly by preventing infiltration of inflammatory cells together with modulating gut microbiota.

## 1. Introduction

Seaweeds are an important source of macronutrients and micronutrients, especially in East and South-East Asia as a major part of the traditional diet [1,2]. The *Caulerpa* genus consists of approximately 75 species of tropical to subtropical siphonous green seaweed [3]. Some *Caulerpa* species, such as *Caulerpa cylindracea* and *Caulerpa taxifiolia*, may cause serious environmental damage as invasive species [4]. *Caulerpa lentillifera,* known as “sea grapes” [5], grown commercially in ponds and consumed in the Philippines, Indonesia and Vietnam, contains proteins, minerals, dietary fibre, vitamins, and saturated and unsaturated fatty acids [6]. Foods containing these components have been investigated for the prevention or reversal of metabolic syndrome [7], defined as a clustering of cardiometabolic risk factors such as obesity, dyslipidaemia, hypertension, fatty liver and glucose intolerance [8].

Decreases in these cardiometabolic risk factors have been reported for *C. lentillifera*, especially improved glucose metabolism and reduced inflammation, which are key symptoms of metabolic syndrome, but no study has examined the responses of *C. lentillifera* on the range of symptoms that constitute diet-induced metabolic syndrome. As examples of studies on aspects of metabolic syndrome, *C. lentillifera* decreased body weight, reduced plasma triglycerides and increased HDL-cholesterol concentrations in high-cholesterol, high-fat diet-fed rats [9]. An ethanolic extract of *C. lentillifera* at 250 mg/kg and 500 mg/kg body weight for six weeks decreased fasting blood glucose concentrations in oral glucose tolerance test and intraperitoneal insulin tolerance test in a genetic model of obesity (C57BL/KsJ-*db*/*db* mice). Furthermore, dosage with 500 mg/kg *C. lentillifera* extract decreased hepatic glycogen content by 54.8% [10]. Polysaccharide fractions purified from *C. lentillifera* enhanced immunostimulatory activity, which increased the proliferation, phagocytosis, nitric oxide production and acid phosphatase activity of macrophages [11]. Reduced inflammation may be due to enhanced immunostimulatory activity through increased production of short-chain fatty acids and gut microbiota diversity and composition of gut microbiota communities in immunosuppressed BALB/c mice by interacting with immune cells and enterocytes in regulating and maintaining the normal function of the innate and adaptive immune system [12]. These immune responses were correlated with improved growth in *Lactobacillus*, *Coriobacteriaceae*, *Ruminococcaceae*, *Clostridium XVIII* and *Helicobacter* and suppressed growth of *Bacteroides*, *Barnesiella* and *Lachnospiraceae* [12].

The aim of this study was to determine whether diet-induced changes in cardiovascular, liver and metabolic responses can be attenuated by chronic dietary intervention with sun-dried whole *C. lentillifera*. We chose this *Caulerpa* species as a controlled aquaculture study reported an increased yield of 2 kg fresh weight/week compared to *C. racemosa*, which yielded <0.5 kg fresh weight/week [5,13]. We sourced *C. lentillifera* from Vietnam, because it is produced there as a commercial product. A validated diet-induced rat model of metabolic syndrome that closely mimics the symptoms of human metabolic syndrome [14] was used for this study. We measured systolic blood pressure, left ventricular diastolic stiffness, inflammatory cells and collagen deposition as cardiovascular responses; plasma liver enzyme activities, liver inflammatory cells and fat vacuoles as liver responses; body weight and composition; total cholesterol and triglyceride concentrations; glucose and insulin tolerance as metabolic responses as well as gut microbiota composition. We hypothesised that increased intake of insoluble fibre with *C. lentillifera* supplementation for the last eight weeks of the protocol will reverse the changes in these parameters induced by the high-carbohydrate, high-fat diet.

## 2. Results

### 2.1. Caulerpa lentillifera Composition

The *C. lentillifera* powder contained (in % dry weight) 16% moisture, 44% carbohydrate, 14% lipid, 7% protein and 17.5% total dietary fibre including 16.6% insoluble fibre. The predominant elements in the *C. lentillifera* biomass were 13.1% Na, 1.1% Mg, 0.81% Ca and 0.67% S (Table 1).

### 2.2. Physiological Variables

**Figure 1:**
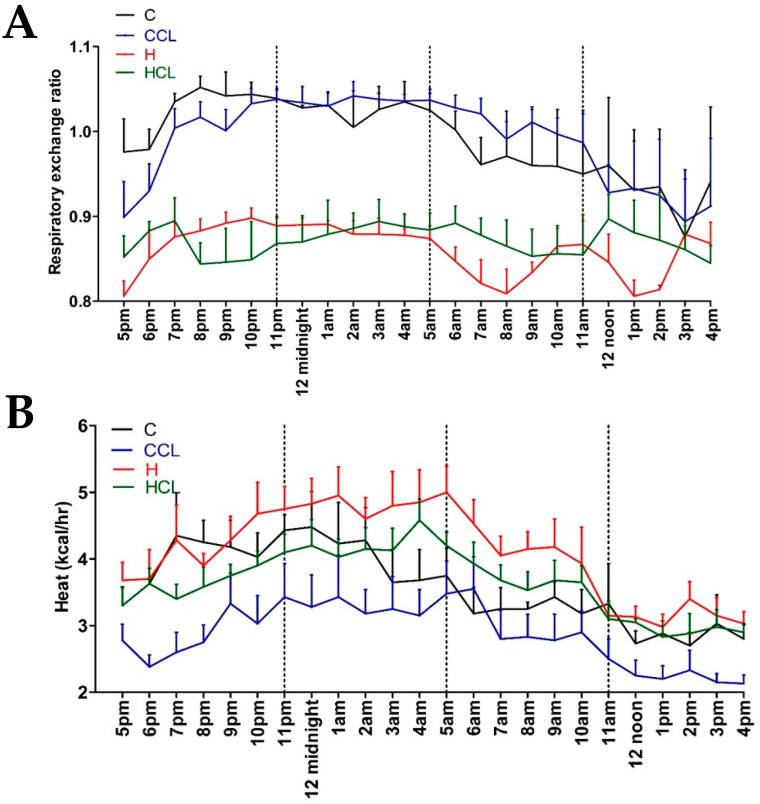
The 24 h indirect calorimeter Oxymax data. (**A**) Respiratory exchange ratio and (**B**) heat production in corn starch diet-fed rats (C), corn starch diet-fed rats supplemented with 5% *Caulerpa lentillifera* (CCL), high-carbohydrate, high-fat diet-fed rats (H) and high-carbohydrate, high-fat diet-fed rats supplemented with 5% *Caulerpa lentillifera* (HCL).

After 16 weeks, the body weight of high-carbohydrate, high-fat diet-fed rats (H) was higher than corn starch diet-fed rats (C); body weight of H rats supplemented with *C. lentillifera* (HCL) was lower than H rats (Table 2). Lean mass was unchanged in all groups (Table 2). Fat mass was highest in H rats followed by HCL, C and C rats supplemented with *C. lentillifera* (CCL) (Table 2). Food intake was higher in C rats compared to H rats. CCL rats had similar food intake as C rats whereas HCL rats ate less food than H rats. C and CCL rats had lower energy intakes than H and HCL rats. CCL and HCL rats had higher water intakes than C and H rats during intervention period (Table 2). C rats had a lower Respiratory Exchange Rate (RER) (Figure 1A) and heat production (Figure 1B) compared to H rats, while HCL rats were lower than H rats. C and CCL rats had lower RER values during the daytime.

Total abdominal fat was highest in H rats followed by HCL, C and CCL rats (Table 2). Plasma triglyceride concentrations were higher in H and HCL rats compared to C and CCL rats. Plasma total cholesterol concentrations were highest in H rats and similar in C, CCL and HCL rats (Table 2). Plasma non-esterified fatty acids were highest in H rats followed by HCL, C and CCL rats. C rats had lower basal blood glucose concentrations compared to H rats. Intervention did not reduce basal blood glucose concentrations (Table 2). The blood glucose area under the curve was not different between groups (Table 2).

**Figure 2:**
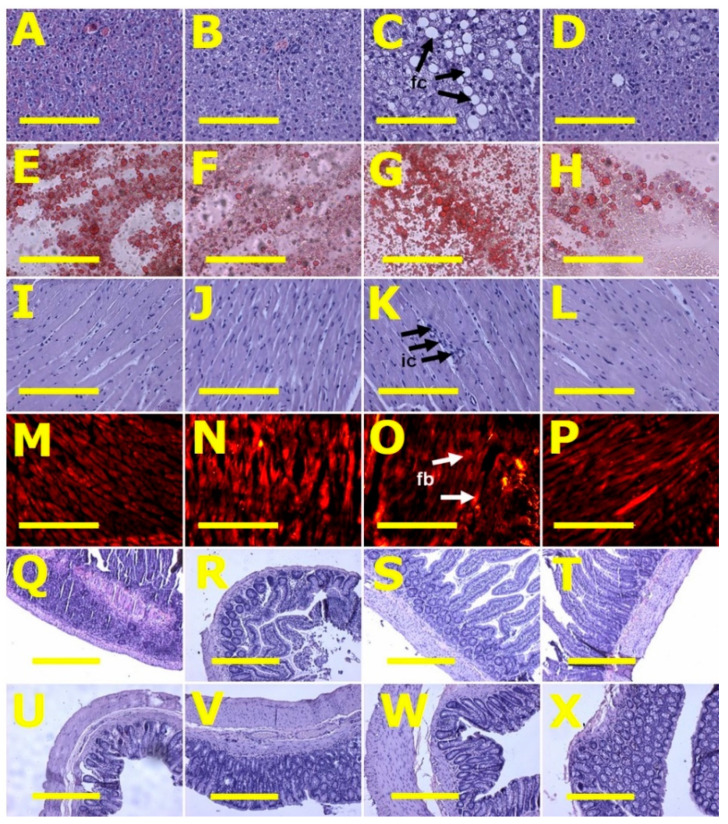
Liver fat structure using haematoxylin and eosin stain (**A**–**D**) and oil red O stain (**E**–**H**); left ventricular structure—heart inflammation (**I**–**L**) using haematoxylin and eosin stain and heart fibrosis (**M**–**P**) for collagen using picrosirius red staining; ileum (**Q**–**T**) and colon (**U**–**X**) structure using haematoxylin and eosin stain in corn starch diet-fed rats (**A**,**E**,**I**,**M**,**Q**,**U**), corn starch diet-fed rats supplemented with *Caulerpa lentillifera* (**B**,**F**,**J**,**N**,**R**,**V**), high-carbohydrate, high-fat diet-fed rats (**C**,**G**,**K**,**O**,**S**,**W**) and high-carbohydrate, high-fat diet-fed rats supplemented with *Caulerpa lentillifera* (**D**,**H**,**L**,**P**,**T**,**X**). Fat cells = fc; inflammatory cells = ic; fibrosis = fb. Scale bar is 200 μm for (**A**–**P**) (20×) and 100 μm for (**Q**–**X**) (10×).

After eight weeks, systolic blood pressure of H diet-fed rats (H and HCL) was higher than C diet-fed rats (C and CCL) (Table 2). Systolic blood pressure in H rats was higher at 16 weeks than in C rats. HCL rats had decreased systolic blood pressure compared to H rats (Table 2). Left ventricular diastolic stiffness was higher in H rats compared to C rats. HCL rats showed normalised left ventricular diastolic stiffness (Table 2). Left ventricular weights with septum and right ventricular wet weights were similar among all groups (Table 2). Left ventricles from H rats showed infiltration of inflammatory cells and increased collagen deposition whereas these changes were not seen in left ventricles from C rats (Figure 2). Left ventricles from HCL rats showed decreased infiltration of inflammatory cells (Figure 2) and decreased collagen deposition compared to H rats (Figure 2). Livers from H rats showed increased fat deposition and infiltration of inflammatory cells compared to livers from C rats (Figure 2). Livers from HCL rats had reduced fat deposition compared to H rats (Figure 2). Plasma activities of transaminases (ALT and AST) were not different among the groups (Table 2).

### 2.3. Gut Structure and Microbiota

Histology of ileum and colon did not show any structural abnormalities in the experimental groups demonstrated by normal crypt depth, villi length and goblet cells and lack of inflammatory cell infiltration (Figure 2).

The gut microbiota was here defined as the collective bacteria in the rat colon. After quality filtering, there were a total of 788,078 bacteria 16S rRNA gene sequences and these were clustered into 1282 zero-radius operational taxonomic unit (zOTUs). The calculated rarefaction curves based on rarefied and unrarefied data as well as Good’s coverage of 99.69 ± 0.08% showed that the bacterial community structure was almost fully recovered by the surveying effort.

**Figure 3:**
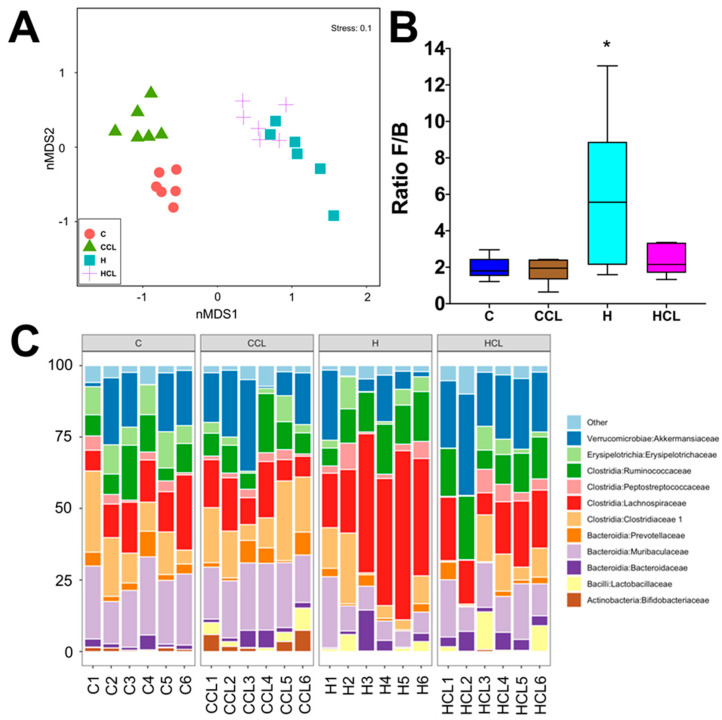
(**A**) Multidimensional scaling (MDS) plot of bacterial community structure of faecal samples from different feeding regimes; (**B**) Effect of supplementation of diet (C or H) with *Caulerpa lentillifera* on the ratio of Firmicutes and Bacteroidetes (F/B) abundances in rat faecal samples. Statistical analysis performed using ANOVA with Tukey’s post hoc test for multiple comparisons, * *p* < 0.05; and (**C**) Taxonomic profiles of bacterial communities shown at the family level of all faecal samples. C, corn starch diet-fed rats; CCL, corn starch diet-fed rats supplemented with *Caulerpa lentillifera*; H, high-carbohydrate, high-fat diet-fed rats; HCL, high-carbohydrate, high-fat diet-fed rats supplemented with *Caulerpa lentillifera*.

Diet and seaweed supplement both affected the overall bacterial community structure based on Bray–Curtis dissimilarity (Figure 3A, Supplementary Table S1; PERMANOVA, both *p* = 0.0001), and there was an interaction between the two factors (Supplementary Table S1; PERMANOVA, *p* = 0.001). There were pairwise differences between the C and H groups indicating an effect of basal feed on the bacterial community structure (*p* = 0.0026). C rats and CCL rats had lower ratios of Firmicutes to Bacteroidetes (F/B ratio) compared to H and HCL rats (Figure 3B). *Caulerpa lentillifera* supplementation reduced the F/B ratio under the H diet. There was no difference in Shannon’s diversity and richness between the four groups (Supplementary Figure S1). The addition of *C. lentillifera* changed the bacterial communities (CCL, *p* = 0.0028; HCL, *p* = 0.0095). Bacterial communities in the CCL group were more variable compared to the C group (Figure 3C, Supplementary Table S1; PERMDISP; *p* = 0.022).

### 2.4. Taxonomic Structure of the Bacterial Communities

The most abundant bacterial classes found in the faecal samples for different treatment groups were Actinobacteria, Bacteroidia, Bacilli, Clostridia, Erysipelotrichia and Verrucomicrobia (Supplementary Figure S2). Other bacterial classes, including Coriobacteriia, Melainabacteria, Deferribacteres, Saccharimonadia, Alphaproteobacteria, Deltaproteobacteria, Gammaproteobacteria and Mollicutes, were observed at lower abundance levels (<1%) in some (but not all) faecal samples.

The relative abundance of bacteria from the class Actinobacteria and Erysipelotrichia was higher in C and CCL rats (Actinobacteria: C, 1.02%; CCL, 3.38%; *p* > 0.05; Erysipelotrichia: C, 9.35%; CCL, 3.57%; *p* > 0.05) compared to H and HCL rats (Actinobacteria: H, 0.02%; HCL, 0.16%; Erysipelotrichia: H, 4.35%; CCL, 2.92%; *p* > 0.05). There was an increase in the relative abundance of bacteria from the class Bacteroidia in C and CCL rats (C, 29.68%; CCL, 29.05%; *p* < 0.01) compared to H and HCL rats (H, 17.15%; HCL, 23.03%) (Supplementary Figure S2). The relative abundance of bacteria from the class Bacilli in H rats (2.28% to 4.28%; *p* > 0.05) was increased compared to C rats (0.59% to 3.28%). Similarly, the relative abundance of bacteria from class Clostridia in H rats (66.31%; *p* < 0.0001) was increased compared to the other groups (C, 43.65%; CCL, 42.45%; HCL, 43.78%) (Supplementary Figure S2). In addition, the relative abundance of bacteria from the class Verrucomicrobiae in HCL rats (24.33%) was increased compared to C rats (13.98%, *p* = 0.0347) and H rats (8.90%, *p* = 0.0004) (Supplementary Figure S2).

Analysis of the bacterial community structure at the family level showed that *Bifidobacteriaceae* (class Actinobacteria), *Bacteroidaceae* (class Bacteriodia), *Muribaculaceae* (class Bacteriodia), *Prevotellaceae* (class Bacteroidia), *Lactobacillaeceae* (class Bacilli), *Clostridiaceae 1* (class Clostridia), *Lachnospiraceae* (class Clostridia), *Peptostreptococcaceae* (class Clostridia), *Ruminococcaceae* (class Clostridia), *Erysipelotrichaceae* (class Erysipelotricia) and *Akkermansiacaeae* (class Verrucomicrobia) were found to be most dominant in the faecal samples (Figure 3C). The relative abundance of bacteria from family *Ruminococcaceae* was reduced in C rats (10.17% to 10.23%; *p* > 0.05) compared to H rats (13.45% to 14.06%). Similarly, a high abundance of bacteria from the family *Lachnospiraceae* was detected in H rats (39.07%, *p* < 0.0001) compared to HCL rats (17.80%) and C rats (13.20% to 15.31%, *p* < 0.0001). The abundance of bacteria from family *Muribaculaceae* was increased in C rats (20.71% to 22.42%, *p* < 0.01) compared to H rats (10.29% to 14.56%), while an increase in the abundance of bacteria from family *Lactobacillaceae* was observed in H rats (1.98% to 4.14%; *p* > 0.05) samples compared to C rats (19.9% to 21.60%) (Figure 3C). Similarly, there was an increase in the relative abundance of bacteria from family Clostridiaceae 1 in C rats compared to H rats, and this difference was more pronounced for samples supplemented with *C. lentillifera* (CCL, 16.45%; HCL, 7.51%; *p* = 0.0285). Moreover, higher abundance of bacteria from family *Bacteroidaceae* was observed for H rats (3.83% to 4.23%, *p* > 0.05) compared to C rats (2.17% to 3.04%) (Figure 3C). In addition, an increase in the relative abundance of bacteria from family *Akkermansiaceae* was detected in H rats supplemented with *C. lentillifera* (24.33%) compared to control samples from both diets (C, 13.98%, *p* = 0.0075; H, 8.90%, *p* < 0.0001) (Figure 3C).

Analysis of the bacterial community structure at the genus level showed that *Bifidobacterium* (family *Bifidobacteriaceae*), *Bacteroides* (family *Bacteroidaceae*), unclassified *Muribaculaeceae*, *Alloprevotella* (family *Prevotellaceae*), *Prevotellaceae UCG-001* (family *Prevotellaceae*), *Lactobacillus* (family *Lactobacillaceae*), *Clostridium sensu stricto 1* (family *Clostridiaceae*), *Lachnospiraceae NK4A136 group* (family *Lachnospiraceae*), *Roseburia* (family *Lachnospiraceae*), unclassified *Lachnospiraceae*, *Romboutsia* (family *Peptostreptococcaceae*), unclassified *Peptostreptococcaceae*, *Ruminiclostridium 9* (family *Ruminococcaceae*), *Ruminiclostridium UCG-014* (family *Ruminococcaceae*), unclassified *Ruminococcaceae*, *Turicibacter* (family *Erysipelotrichaceae*) and *Akkermansia* (family *Akkermansiaceae*) were found to be most dominant in the faecal samples (Supplementary Figure S3).

### 2.5. Multivariate Analysis of Physiological and Microbiota Data

A total of 23 physiological parameters were assessed and included for analysis (Supplementary Table S2). These included body weight, fat mass, lean mass, water intake, food intake, energy intake, feed efficiency, left ventricle with septum wet weight, right ventricle wet weight, retroperitoneal fat, omental fat, epididymal fat, total abdominal fat, liver wet weight, kidney wet weight, spleen wet weight, plasma non-esterified fatty acids, plasma triglycerides, systolic blood pressure, oral glucose tolerance area under the curve, oral glucose tolerance 120 min blood glucose concentrations, plasma aspartate transaminase activity and plasma alanine transaminase activity for rats fed with the C and H diets and supplemented with *C. lentillifera* (Supplementary Figure S4). Additional results for gut microbiota analysis are presented in Supplementary Table S3, Supplementary Table S4 and Supplementary Table S5. The physiological variables most often related to changes in the gut microbiota were the oral glucose tolerance test (120 min glucose concentration and area under the curve), liver wet weight, retroperitoneal, epididymal and total abdominal fat.

## 3. Discussion

Aquaculture is a sustainable method to grow tropical seaweeds for human food production and for biodiversity enrichment with the potential to enhance fisheries [15]. Fresh seaweed can be used in salads, fruit and vegetable juices, re-hydrated seaweed can be used in a variety of dishes with rice and beans, and dried seaweed can be used as substitute for wheat and maize flours for baking and cooking [15]. There are a variety of recipes available for *C. lentillifera*, with popular dishes including green caviar, named due to its similar appearance to fish eggs. *Caulerpa* species are traditionally used in the Indo-Pacific area as fresh vegetables due to their palatable taste, their availability, their nutritional properties and people’s general awareness of natural products [16,17]. *Caulerpa lentillifera* has been cultivated in 1 m^2^ trays to control quality and increase the accessibility of the crop during harvesting periods [17].

*Caulerpa lentillifera* was chosen over other *Caulerpa* species such as *C. racemosa* for this study, because of its better growth in Australian tropical waters, potential commercial role and lower accumulation of toxic metals [6,13]. The powdered seaweed was bought from a supplier in Vietnam, because this is currently a commercial product and thus represents the realistic seaweed market. Unlike *C. racemosa* [18], *C. lentillifera* did not accumulate potentially toxic minerals, which may have been present in the water in which it grows, including lead, mercury and arsenic. *C. lentillifera* supplementation improved cardiometabolic risk factors in HCL rats by decreasing body weight by ~20% compared to H rats, reducing systolic blood pressure, reducing left ventricular diastolic stiffness constant, and reducing plasma total cholesterol and non-esterified fatty acid concentrations. The gut microbiota of HCL rats was different to H rats, and closer to CCL rats. Chloroform and water extracts from *C. racemosa and C. lentillifera* demonstrated an antibacterial effect against the pathogenic methicillin-resistant *Staphylococcus aureus* [19]. These extracts and whole seaweeds may thus directly change the gut microbiota of rats and humans.

Compounds of *C. lentillifera* include carbohydrates such as sulphated polysaccharides, sterols and proteins [20]; the intervention in this project contained similar macronutrient content to the literature [21] except for a 5–6-fold higher lipid content. *Caulerpa* species contain minerals with Na at the highest concentration followed by K, Ca and Mg [21]. In addition, *Caulerpa* species contain secondary metabolites including flavonoids, caulerpin, caulerpenyne [22] and siphonaxanthin with possible bioactivity [23]. Siphonaxanthin is a carotenoid found in green algae such as *C. lentillifera* and has been shown to suppress inflammation induced by advanced glycation end-products, which is a key contributor to the pathogenesis of atherosclerosis [24]. Caulerpin is a bis-indole alkaloid isolated from the marine green seaweed *Caulerpa* and red seaweed *Chondria armata* [25]. It is potentially bioactive with a wide range of therapeutic activities including anti-diabetic [26], anti-inflammatory and anti-nociceptive properties [27]. This study investigated the whole seaweed and it is plausible that the physiological and metabolic responses in the rats were produced by the combination of bioactive compounds.

Several studies have reported the health benefits of seaweed extracts [28], which may be attributed to their unique nutritional profile of polyunsaturated fatty acids, pigments, trace minerals and polysaccharides [13]. Hypotheses surrounding mechanisms for green algae in improving health include prebiotic effects from their high fibre content. *Caulerpa lentillifera* polysaccharides are sulphated and hence similar to the bioactive carrageenans from red seaweed, which we and others have shown to be effective against metabolic syndrome using the same rat model [29,30,31]. Sulphated polysaccharides from *Caulerpa* species consist of a complex and heterogeneous structure of repetitive sugars units [32]. It is likely that these compounds are involved in mechanical, ionic and osmotic regulation, favouring the survival of these organisms in the marine environment [32]. *Caulerpa* polysaccharides undergo minimal digestion in the stomach, but rather are fermented by colonic bacteria [33], hence meeting the definition of prebiotics [34]. Health benefits of prebiotics include decreased blood pressure and body weight [35,36] similar to the responses from the current study. Using the same rat model of diet-induced obesity, a prebiotic mixture of inulin and oligofructose was an effective dietary fibre reducing body weight gain, plasma concentrations of free fatty acids and triglycerides, and systolic blood pressure, and attenuating inflammatory cell infiltration in the heart and liver [37]. *Caulerpa lentillifera* from the current study contained 17.5% total dietary fibre of which 16.6% was insoluble fibre which may have resulted in an increased colonic production of short-chain fatty acids (SCFAs) including acetic, propionic and butyric acids [38]. Almost all of the fibre was insoluble which is not converted to energy and improves faecal bulking and thus increases satiety [39]. In a previous study, we suggested that increasing soluble fibre intake with inulin and oligofructose improved signs of the metabolic syndrome by decreasing gastrointestinal carbohydrate and lipid uptake [37]. This is extended in the current study to show that the insoluble fibre component of *C. lentillifera* is the most likely bioactive compound mediating the improved metabolic, liver and cardiovascular responses.

Chronic metabolic disorders including obesity, type 2 diabetes and cardiovascular disease are characterised by low-grade inflammation [40]. There is evidence that foods containing anti-inflammatory compounds help decrease obesity [7]. Consumption of a high-fat diet leads to increased concentrations of gut inflammatory cytokines such as TNF-α, IL-1β and IL-12 which are related to weight gain, adiposity, and increased plasma insulin and glucose concentrations [41]. An intervention that reduces gut and systemic inflammation may lead to decreased obesity-associated conditions such as metabolic syndrome. Chronic inflammation in adipose tissue likely plays a crucial role in the development of obesity-associated insulin resistance [42]. The role of innate lymphoid cells in mediating obesity-associated inflammation has been investigated [43,44]. *Caulerpa* species have shown anti-obesity, anti-diabetic, anti-hypertensive, anti-inflammatory, anti-nociceptive and anti-tumour responses [25]. In the present study, the reduced heart and liver inflammation may be due to an immunostimulatory effect demonstrated in other studies with *C. lentillifera* polysaccharides where there has been enhanced immunostimulatory activity in immunosuppressed mice and modulated gut microbiota [12]. *Caulerpa lentillifera* polysaccharides increased synthesis and secretion of cytokines including IL-6, TNF-α, IL-1β and nitric oxide [45]. This demonstrates the potential of this seaweed to improve inflammatory conditions such as obesity.

Beneficial physiological effects, including cardiovascular improvements such as decreased blood pressure, are controlled by the gut microbiota and mediated by SCFAs [46], while SCFAs promote a lean and healthy phenotype [47,48]. Polysaccharides exert their action through a wide range of mechanisms including selective fermentation, lowering the gut pH, faecal bulking, preventing gut colonisation by pathogens, controlling putrefactive bacteria, and therefore reducing the host’s exposure to toxic metabolites [49]. These effects are likely due to dietary fibre increasing SCFA production as SCFAs are used as an energy source by selected gut microbiota. SCFAs decrease luminal pH, improve calcium and magnesium absorption, reduce potential pathogenic bacteria and act as an energy source for epithelial cells [50,51].

There is scarce information on global seaweed consumption, with data from Japan the most reliable. Daily seaweed consumption per person in Japan remained relatively consistent at 4.3 g/day in 1955 and 5.3 g/day in 1995 [52]. In the current study, HCL rats consumed around 1 g/day *C. lentillifera* for the final eight weeks of the protocol. Using the Reagan-Shaw calculation for rat-to-human scaling [53], humans would need to consume 5.8 g/day for an equivalent dose to the current study, which is realistic based on reported seaweed intake in Japan.

The moisture content of fresh seaweed is very high, up to 94% of the biomass [28]. Consequently, in this study we used dried biomass to determine the effects of the non-water-soluble compounds, such as fibre for their prebiotic effects and pigments such as chlorophyll and *β*-carotene for antioxidant or anti-inflammatory effects. In mice, 5 mg/kg body mass of chlorophyllin derived from chlorophyll in the drinking water for eight weeks attenuated intestinal and hepatic inflammation and ameliorated liver fibrosis [54] possibly by inhibiting the NF-κB pathway and modulating gut microbiota. Approximately 59% of gut bacteria were Bacteroidetes, while 14% were Firmicutes in both control and chlorophyllin-supplemented mice. In contrast, in the fibrotic mice, the population was changed, showing decreased Bacteroidetes as 29% and increased Firmicutes as 40% of total bacterial content [54]. In the current study, *C. lentillifera* also clearly modulated the gut microbiota and decreased the F/B ratio, which may be a mechanism for the physiological and metabolic improvements in HCL rats. Using the whole seaweed, we suggest that all components are working together for the observed responses in the rats.

## 4. Materials and Methods

### 4.1. Caulerpa lentillifera Source and Elemental Composition Analysis

*Caulerpa lentillifera* was purchased from Viet Delta Corporation, Ho Chi Minh City, Vietnam. A total of 5 kg of sun-dried *C. lentillifera* was transported to the University of Southern Queensland, Toowoomba, QLD, Australia, in vacuum-sealed bags containing silica desiccant. Soluble and insoluble dietary fibre were analysed by a commercial laboratory (Symbio Laboratories, Eight Mile Plains, QLD, Australia). For elemental composition analysis, approximately 0.1 g of *C. lentillifera* biomass was placed in a digestion vessel and 3.5 mL of double-distilled HNO_3_ and 0.5 mL of super-pure H_2_O_2_ was added. The mixture was left in a fumehood for approximately 1 h before it was loaded into a Berghof Speedwave microwave digestion system for sample digestion. Then the solution was quantitatively transferred into a 50 mL volumetric flask and filled up to the mark using Milli-Q water. This solution was analysed for Al, Ca, Mg, K, Na, P and S by an Agilent 5100 ICP-OES and the other elements were analysed by a Varian 820 ICP-MS (Advanced Analytical Centre, James Cook University, Townsville, QLD, Australia).

### 4.2. Rats and Diets

All experimental protocols were approved by the Animal Ethics Committee of the University of Southern Queensland (approval number 16REA014) under the guidelines of the National Health and Medical Research Council of Australia. Male Wistar rats (8–9 weeks old; 338 ± 1 g, *n* = 48) were obtained from the Animal Resource Centre, Murdoch, WA, Australia. Rats were individually housed in a temperature-controlled (21 ± 2 °C), 12 h light/dark conditions with free access to food and water. Rats were randomly allocated to four groups, each of 12 rats. Two groups were fed either corn starch or high-carbohydrate, high-fat diets (C and H, respectively) [14] for the full 16 weeks. The other two groups received C and H diets for eight weeks and then received 5% dried *C. lentillifera* mixed in the food for the final eight weeks (CCL and HCL, respectively). Daily body weights in grams and daily food and water intakes in grams were measured. Daily food intake was used to calculate both seaweed dosage and caloric intake (with contribution from fructose in water for H and HCL rats).

### 4.3. Rat Measurements

An anaesthesia machine (Mediquip, Loganholme, QLD, Australia) was used to induce light sedation to immobilise rats for dual-energy X-ray absorptiometry and non-invasive systolic blood pressure measurements. Rats were pre-oxygenated with 2 L/min for three minutes prior to 5% isoflurane induction vaporised in 1.5 L/min. Rats were placed in a nose mask and maintained with 1.5% isoflurane in 1.5 L/min O_2_ for the duration of each procedure. Dual-energy X-ray absorptiometry, non-invasive systolic blood pressure, abdominal circumference, oral glucose and insulin tolerance tests, indirect calorimetry, plasma measurements, isolated Langendorff heart preparation and organ weights were performed as described [14,55]. Rats were allowed to acclimate in the metabolic chamber for approximately 30 min before measurements started. Euthanasia followed by heparin injection, blood collection, centrifugation, storage and then isolated Langendorff heart preparation and measurements, plasma measurements, organ weights and histological analyses were performed as described [14].

Immediately after euthanasia and organ removal, two or three faecal pellets were collected from the colon of rats and stored at −80 °C in nuclease-free tubes.

### 4.4. Gut Microbiota Analysis

Total microbial community DNA was extracted from faecal samples using the DNeasy Powersoil Kit (Qiagen Australia, Chadstone, VIC, Australia) following the manufacturer’s instructions [56]. The bacterial gut microbiota was then characterised by amplifying and sequencing the V_3_–V_4_ regions of 16S rRNA gene using primer 341F (TCGTCGGCAGCGTCAGATGTGTATAAGAGACAGCCT ACGGGNGGCWGCAG) and 785R (GTCTCGTGGGCTCGGAGATGTGTATAAGAGAC AGGACTACHVGGGTATCTAATCC).

The amplification reaction mixture (50 μL total volume per sample) consisted of Econotaq® PLUS GREEN 2× Master Mix (Astral Scientific, Gymea, NSW, Australia) (25 μL), Ambion® nuclease-free water (17 μL), the primer pair 341F and 785R (1.5 μL of each; 10 μM) and DNA template (5 μL). The PCR program consisted of an initial denaturation at 94 °C (2 min), followed by 35 cycles of denaturation at 94 °C (30 s), annealing at 55 °C (30 s), extension at 72 °C (40 s) and a final extension at 72 °C (7 min). Paired-end sequencing (2 × 300 bp) of the resulting 16S rRNA gene amplicons was performed at the Ramaciotti Centre for Genomics, University of New South Wales on an Illumina MiSeq platform following the MiSeq System User Guide [57].

Sequence data were initially quality filtered and trimmed using Trimmomatic version 0.36 truncating reads if the quality dropped below 20 in a sliding window of 4 bp [58]. USEARCH version 11.0.667 [59] was used for further processing [60] to merge and quality-filter sequencing reads, excluding reads with <250 or >550 nucleotides, in addition to reads with more than one ambiguous base or an expected error of more than 1. Filtered sequences were denoised and clustered into unique sequences (zero-radius operational taxonomic units; zOTUs) using the UNOISE algorithm [61] implemented in USEARCH. Chimeric sequences were removed de novo during clustering and subsequently in reference mode using UCHIME [62] with the SILVA database (SILVA SSURef 132 NR) as a reference [63]. zOTUs were then taxonomically classified by BLASTN [64] against the SILVA database. All non-bacterial zOTUs were removed along with non-BLAST aligned and singleton zOTUs. Finally, processed sequences were mapped on zOTU sequences to calculate the distribution and counts of each zOTU in every sample. Only zOTUs occurring in more than two samples were considered for further statistical analysis.

### 4.5. Statistical Analysis

All data are presented as the mean ± standard error of the mean (SEM). Metabolic and physiological results were tested for variance using Bartlett’s test and variables that were not normally distributed were transformed using log10 function prior to statistical analyses. Data from the four groups were tested by two-way analysis of variance. When the interactions and/or the main effects were significant, means were compared using the Newman–Keuls multiple comparison post hoc test. Where transformations did not result in normality or constant variance, a Kruskal–Wallis non-parametric test was performed. A *p* value of <0.05 was considered as statistically significant. All statistical analyses were performed using Prism version 5.00 for Windows (GraphPad Software, San Diego, CA, USA).

For microbiota results, rarefaction curves were generated using the *rarecurve* function in *vegan* [65] and used to determine if a complete representation of the sample’s microbiota had been achieved given the sequencing effort. Prior to further analysis, the numbers of sequences were standardised across samples to account for different sequencing depths by randomly subsampling each sample to the lowest number of sequences counts obtained for any given sample (i.e., 23,952 counts). Bacterial alpha-diversities (i.e., zOTU richness and Shannon’s diversity) were calculated in R (version 3.5.3) using the *rrarefy* function in the *vegan* package for community ecology analysis [65]. A one-way ANOVA test in GraphPad Prism 8.0.2 (San Diego, CA, USA) followed by Tukey’s pairwise comparisons test was used to determine the significance between the different groups, a *p* value of <0.05 was considered as statistically significant.

For multivariate analysis of bacterial communities, OTU tables were imported into PRIMER [66] to compare the community structure (i.e., relative abundance data). Bray–Curtis similarity coefficients were calculated using square root-transformed zOTU abundances and the resulting similarity matrix was visualised using non-metric, multidimensional scaling (nMDS). Permutational multivariate analysis of variance (PERMANOVA) [67] with 9999 random mutations was used to test the effect of treatment on bacterial communities in rat faecal samples. ‘Treatment’ was set with corn starch diet (C), corn starch diet supplemented with *C. lentillifera* (CCL), high-carbohydrate, high-fat diet (H) and high-carbohydrate, high-fat diet supplemented with *C. lentillifera* (HCL) as fixed factors.

## 5. Conclusions

*Caulerpa lentillifera* as a functional food may reverse the changes in metabolic syndrome since supplementation reduced body fat mass, systolic blood pressure, diastolic stiffness constant, and plasma total cholesterol and non-esterified fatty acid concentrations in this diet-induced rat model of human metabolic syndrome. We suggest that these biological responses are based on the prebiotic responses to the sulphated polysaccharides as the major component of the insoluble fibre in this seaweed, augmented by the actions of other macronutrients, secondary metabolites and minerals. The gut microbiota in HCL rats was modulated towards healthy control rats which may have provided the metabolic syndrome improvements. In addition, the presence of prebiotics may decrease nutrient absorption in the intestine. Further investigation into potential mechanisms of action may provide additional evidence for the insoluble fibre from *C. lentillifera* as a functional food in other inflammatory-related diseases. Large-scale cultivation can provide additional uses for this seaweed such as animal food, as broad-spectrum crop fertilisers, to remediate waste-waters and to provide extracts for biotechnological applications [68]. Although currently only widely consumed in South-East Asia and the Pacific, there are good reasons for Western countries to cultivate and consume *C. lentillifera* for its potential health benefits.

## Figures and Tables

**Table 1:** Elemental composition of dried *Caulerpa lentillifera* biomass.

Element	Concentration (mg/kg)
Aluminium (Al)	744 ± 8
Arsenic (As)	≤1
Barium (Ba)	4.75 ± 0.19
Boron (B)	21.7 ± 0.5
Cadmium (Cd)	1.14 ± 0.03
Calcium (Ca)	8137 ± 73
Chromium (Cr)	3.3 ± 0.5
Cobalt (Co)	1.35 ± 0.05
Copper (Cu)	2.74 ± 0.3
Iron (Fe)	595 ± 5
Lead (Pb)	2.22 ± 0.08
Magnesium (Mg)	10,663 ± 52
Manganese (Mn)	425 ± 14
Mercury (Hg)	≤1
Molybdenum (Mo)	1.32 ± 0.14
Nickel (Ni)	1.88 ± 0.19
Phosphorus (P)	1073 ± 17
Potassium (K)	1066 ± 546
Selenium (Se)	≤1
Sodium (Na)	130,794 ± 763
Strontium (Sr)	104 ± 3
Sulphur (S)	6733 ± 72
Vanadium (V)	2.46 ± 0.08
Zinc (Zn)	15.2 ± 0.3

Values are the mean ± SD.

**Table 2:** Responses to *Caulerpa lentillifera*.

Variables	C	CCL	H	HCL	*p* Value		
					Diet	Treatment	Interaction
Physiological Variables							
0 week body weight, g	337 ± 1	339 ± 1	338 ± 1	337 ± 1	0.6196	0.6196	0.1408
8 week body weight, g	364 ± 12 ^b^	358 ± 6 ^b^	439 ± 9 ^a^	440 ± 8 ^a^	<0.0001	0.7828	0.6997
16 week body weight, g	411 ± 14 ^c^	389 ± 6 ^c^	573 ± 10 ^a^	468 ± 10 ^b^	<0.0001	<0.0001	0.0002
16 week lean mass, g	316 ± 7	314 ± 5	303 ± 14	303 ± 9	0.2069	0.9155	0.9155
16 week fat mass, g	80 ± 9 ^c^	57 ± 4 ^d^	258 ± 28 ^a^	148 ± 14 ^b^	<0.0001	0.0002	0.0111
8 week lean/fat mass proportion	5.9 ± 0.7 ^b^	10.7 ± 2.6 ^a^	2.6 ± 0.2 ^c^	3.2 ± 1.2 ^c^	0.0007	0.0744	0.1622
16 week lean/fat mass proportion	4.2 ± 0.5 ^b^	6.0 ± 0.7 ^a^	1.3 ± 0.2 ^d^	2.3 ± 0.4 ^c^	<0.0001	0.0060	0.4137
16 week bone mineral content, g	11.9 ± 0.4 ^c^	11.4 ± 0.2 ^c^	17.7 ± 1.1 ^a^	14.3 ± 0.5 ^b^	<0.0001	0.0014	0.0139
16 week bone mineral density, g/cm^2^	0.184 ± 0.004 ^b^	0.171 ± 0.003 ^c^	0.191 ± 0.005 ^a^	0.183 ± 0.004 ^b^	0.0309	0.0179	0.5565
Food intake 0–8 weeks, g/day	37.5 ± 1.4 ^a^	37.6 ± 0.4 ^a^	27.8 ± 1.7 ^b^	25.9 ± 0.7 ^b^	<0.0001	0.4469	0.3984
Food intake 9–16 weeks, g/day	39.7 ± 2.2 ^a^	37.5 ± 0.9 ^a^	29.3 ± 2.5 ^b^	19.3 ± 1.0 ^c^	<0.0001	0.0015	0.0353
Water intake 0–8 weeks, g/day	33.4 ± 2.8	30.2 ± 1.7	31.4 ± 3.1	29.1 ± 0.7	0.5005	0.2346	0.8446
Water intake 9–16 weeks, g/day	25.9 ± 3.3 ^b^	52.6 ± 2.0 ^a^	29.8 ± 2.1 ^b^	50.8 ± 1.2 ^a^	0.6470	<0.0001	0.2173
Energy intake 0–8 weeks, kJ/day	421 ± 16 ^b^	422 ± 4 ^b^	603 ± 34 ^a^	598 ± 25 ^a^	<0.0001	0.9301	0.8953
Energy intake 9–16 weeks, kJ/day	447 ± 24 ^b^	416 ± 10 ^b^	620 ± 46 ^a^	551 ± 21 ^a^	<0.0001	0.0856	0.5074
16 week abdominal circumference, cm	19.0 ± 0.4 ^c^	18.4 ± 0.2 ^c^	23.4 ± 0.4 ^a^	21.1 ± 0.3 ^b^	<0.0001	<0.0001	0.0149
Body mass index, g/cm^2^	0.67 ± 0.02 ^b^	0.63 ± 0.01 ^b^	0.77 ± 0.02 ^a^	0.69 ± 0.01 ^b^	<0.0001	0.0004	0.2126
Retroperitoneal fat, mg/mm	231 ± 15 ^c^	178 ± 13 ^d^	628 ± 68 ^a^	398 ± 37 ^b^	<0.0001	0.0010	0.0320
Epididymal fat, mg/mm	69 ± 6 ^c^	41 ± 4 ^d^	182 ± 32 ^a^	134 ± 16 ^b^	<0.0001	0.0432	0.5865
Omental fat, mg/mm	149 ± 14 ^c^	134 ± 7 ^c^	325 ± 39 ^a^	229 ± 31 ^b^	<0.0001	0.0392	0.1280
Total abdominal fat, mg/mm	450 ± 27 ^c^	353 ± 21 ^d^	1136 ± 135 ^a^	761 ± 77 ^b^	<0.0001	0.0049	0.0876
Visceral adiposity, %	5.1 ± 0.2 ^c^	4.3 ± 0.2 ^d^	9.8 ± 0.8 ^a^	7.7 ± 0.7 ^b^	<0.0001	0.0115	0.2436
Liver wet weight, mg/mm	235 ± 11 ^b^	250 ± 6 ^b^	403 ± 18 ^a^	379 ± 13 ^a^	<0.0001	0.7258	0.1332
Cardiovascular Variables							
8 week systolic blood pressure, mmHg	118 ± 6	120 ± 2	131 ± 2	126 ± 3	0.0083	0.6597	0.3075
16 week systolic blood pressure, mmHg	119 ± 3 ^b^	117 ± 4 ^b^	135 ± 3 ^a^	118 ± 3 ^b^	0.0356	0.0198	0.0617
Left ventricle + septum wet weight, mg/mm	21.5 ± 1.5 ^b^	21.4 ± 1.1 ^b^	25.8 ± 2.0 ^a^	22.5 ± 0.4 ^b^	0.0314	0.1661	0.1917
Right ventricle, mg/mm	4.2 ± 0.7	4.7 ± 0.3	4.5 ± 0.2	4.7 ± 0.3	0.7057	0.3805	0.7057
Left ventricular diastolic stiffness (κ)	21.1 ± 1.5 ^c^	22.1 ± 1.2 ^c^	30.2 ± 0.6 ^a^	27.1 ± 0.8 ^b^	<0.0001	0.3375	0.0649
Left ventricle collagen area, %	7.8 ± 0.7 ^c^	9.1 ± 1.2 ^c^	29.4 ± 2.2 ^a^	22.1 ± 2.8 ^b^	<0.0001	0.2748	0.1482
Metabolic Variables							
Plasma total cholesterol, mmol/L	1.59 ± 0.06 ^b^	1.54 ± 0.05 ^b^	1.73 ± 0.09 ^a^	1.56 ± 0.06 ^b^	0.2368	0.1063	0.3733
HDL-cholesterol, mmol/L	0.93 ± 0.14 ^a^	1.11 ± 0.07 ^a^	0.95 ± 0.09 ^a^	0.70 ± 0.05 ^b^	0.0268	<0.0001	<0.0001
LDL-cholesterol, mmol/L	0.55 ± 0.14	0.80 ± 0.32	0.92 ± 0.08	0.61 ± 0.07	0.7120	0.9020	0.2551
Plasma triglycerides, mmol/L	0.50 ± 0.05 ^b^	0.64 ± 0.10 ^b^	1.15 ± 0.13 ^a^	1.12 ± 0.18 ^a^	<0.0001	0.6603	0.4977
Plasma non-esterified fatty acids, mmol/L	0.68 ± 0.12 ^c^	0.47 ± 0.04 ^d^	2.71 ± 0.29 ^a^	1.61 ± 0.49 ^b^	<0.0001	0.0298	0.1342
Alanine transaminase, U/L	39 ± 5	39 ± 6	50 ± 11	62 ± 12	0.0663	0.5098	0.5098
Aspartate transaminase, U/L	138 ± 20	163 ± 14	174 ± 17	220 ± 45	0.0917	0.1949	0.6989
Liver inflammatory cells, cells/200µm^2^	12 ± 2	13 ± 2	28 ± 2	21 ± 3	0.0001	0.2859	0.1576
Liver fat vacuoles area, µm^2^	11.2 ± 1.9 ^c^	9.2 ± 1.4 ^c^	84.5 ± 2.6 ^a^	45.5 ± 3.1 ^b^	<0.0001	<0.0001	<0.0001
Oral Glucose Tolerance Test							
0 week basal blood glucose, mmol/L	2.8 ± 0.1	2.8 ± 0.1	2.9 ± 0.1	2.8 ± 0.1	0.6196	0.6196	0.6196
0 week area under the curve, mmol/L × min	648 ± 31	641 ± 19	620 ± 44	633 ± 19	0.5526	0.9210	0.7411
8 week basal blood glucose, mmol/L	2.5 ± 0.2	2.5 ± 0.1	3.2 ± 0.1	3.4 ± 0.2	<0.0001	0.5304	0.5304
8 week 120 min blood glucose, mmol/L	4.0 ± 0.1	3.7 ± 0.1	4.8 ± 0.4	5.3 ± 0.1	<0.0001	0.6486	0.0732
8 week area under the curve, mmol/L × min	526 ± 24	566 ± 15	613 ± 23	688 ± 14	<0.0001	0.0052	0.3751
16 week basal blood glucose, mmol/L	2.9 ± 0.2	2.4 ± 0.1	2.8 ± 0.2	2.8 ± 0.1	0.3480	0.1210	0.1210
16 week 120 min blood glucose, mmol/L	3.4 ± 0.1	3.3 ± 0.1	4.7 ± 0.3	4.5 ± 0.1	<0.0001	0.3912	0.7742
16 week area under the curve, mmol/L × mins	478 ± 17	472 ± 11	563 ± 16	571 ± 9	<0.0001	0.9505	0.6643
Insulin Tolerance Test							
8 week 120 min blood glucose, mmol/L	3.3 ± 0.9	2.7 ± 0.5	3.0 ± 0.4	4.3 ± 0.2	0.2531	0.5361	0.0976
8 week area under the curve, mmol/L × min	147 ± 45	144 ± 22	353 ± 40	441 ± 20	<0.0001	0.2122	0.1823
16 week 120 min blood glucose, mmol/L	1.1 ± 0.4	2.3 ± 0.5	3.6 ± 0.3	3.4 ± 0.3	<0.0001	0.1997	0.0752
16 week area under the curve, mmol/L × min	111 ± 11	207 ± 28	356 ± 36	332 ± 28	<0.0001	0.1944	0.0334

Values are presented as the mean ± SEM, *n* = 10–12. Means in a row with superscripts without a common letter differ (a, b, c or d), *p* < 0.05. C, corn starch diet-fed rats; CCL, corn starch diet-fed rats supplemented with *Caulerpa lentillifera*; H, high-carbohydrate, high-fat diet-fed rats; HCL, high-carbohydrate, high-fat diet-fed rats supplemented with *Caulerpa lentillifera*.

## Supplementary Materials

The following are available online at https://www.mdpi.com/2218-1989/10/12/500/s1, Figure S1: Shannon diversity (A) and richness (B) of faecal samples; Figure S2: Taxonomic profiles of bacterial communities shown at the class level of all faecal samples; Figure S3: Taxonomic profiles of bacterial communities shown at the genus level of all faecal samples; Figure S4: Correlation between bacterial community structure (points) and environmental variables (arrows); Table S1: PERMANOVAs based on the Bray–Curtis similarity measure for square root-transformed abundances of all rat faecal samples; Table S2: Correlation between bacterial community structure and physiological parameters (*p* < 0.05); Table S3: Relative abundance of zOTUs affected by diet (ANOVA with *p* adjusted <0.05) between C, CCL, H and HCL rats; Table S4: Relative abundance of zOTUs affected by treatment (ANOVA with *p* adjusted <0.05) between C, CCL, H and HCL rats; Table S5: Taxonomic assignments of the zOTUs strongly correlated with physiological parameters.
