# Supplementary material for: Caulerpa lentillifera (Sea Grapes) Improves Cardiovascular and Metabolic Health of Rats with Diet-Induced Metabolic Syndrome
Source: Metabolites. 2020 Dec 7;10(12):500. doi: 10.3390/metabo10120500 (PMC7762211; doi:10.3390/metabo10120500)
Supplement: Supplementary file 1 [file metabolites-10-00500-s001.pdf]

metabolites

Article

Caulerpa lentillifera (Sea Grapes) Improves Cardiovascular and Metabolic Health of Rats with Diet-Induced Metabolic Syndrome

Ryan du Preez [1,†], Marwan E. Majzoub [2,3], Torsten Thomas [2,3], Sunil K. Panchal [1,‡] and Lindsay Brown [1,4,*]

[1] Functional Foods Research Group, University of Southern Queensland, Toowoomba QLD 4350, Australia; r.dupreez@cqu.edu.au (R.d.P.); S.Panchal@westernsydney.edu.au (S.K.P.)
[2] Centre for Marine Science and Innovation, University of New South Wales, Sydney NSW 2052, Australia; m.majzoub@unsw.edu.au (M.E.M.), t.thomas@unsw.edu.au (T.T.)
[3] School of Biological, Earth and Environmental Sciences, University of New South Wales, Sydney NSW 2052, Australia
[4] School of Health and Wellbeing, University of Southern Queensland, Ipswich QLD 4305, Australia
† School of Health, Medical and Applied Sciences, Central Queensland University, Rockhampton QLD 4701, Australia
‡ School of Science, Western Sydney University, Richmond NSW 2753, Australia
* Correspondence: Lindsay.Brown@usq.edu.au; Tel.: +61-7-3812-6366

Received: 12 October 2020; Accepted: 3 December 2020; Published: date

Supplementary Figures

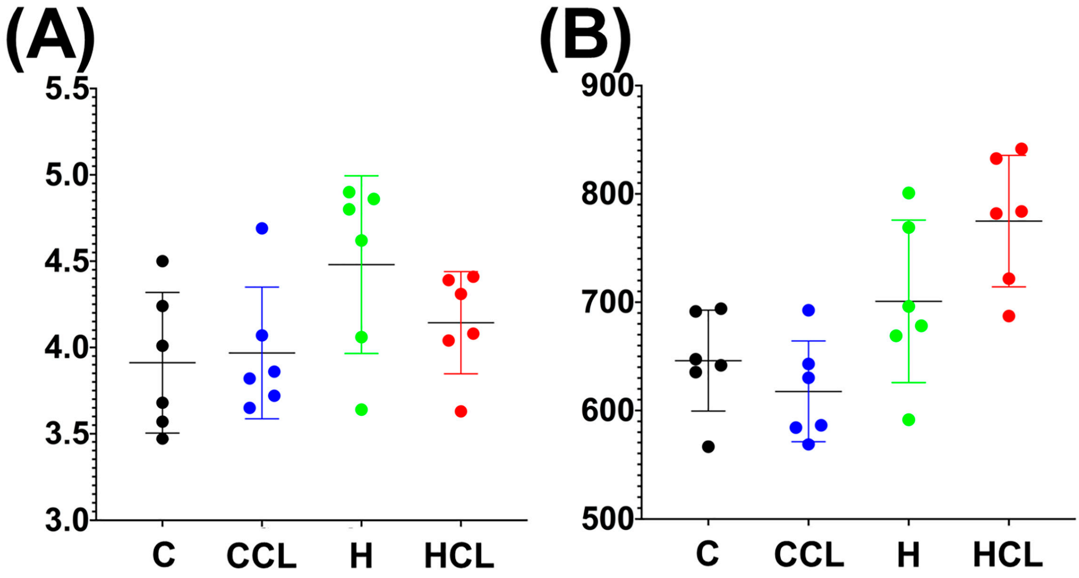

Figure S1. Shannon diversity (**A**) and richness (**B**) of faecal samples. C, corn starch diet-fed rats; CCL, corn starch diet-fed rats supplemented with *Caulerpa lentillifera*; H, high-carbohydrate, high-fat diet-fed rats; HCL, high-carbohydrate, high-fat diet-fed rats supplemented with *Caulerpa lentillifera*.

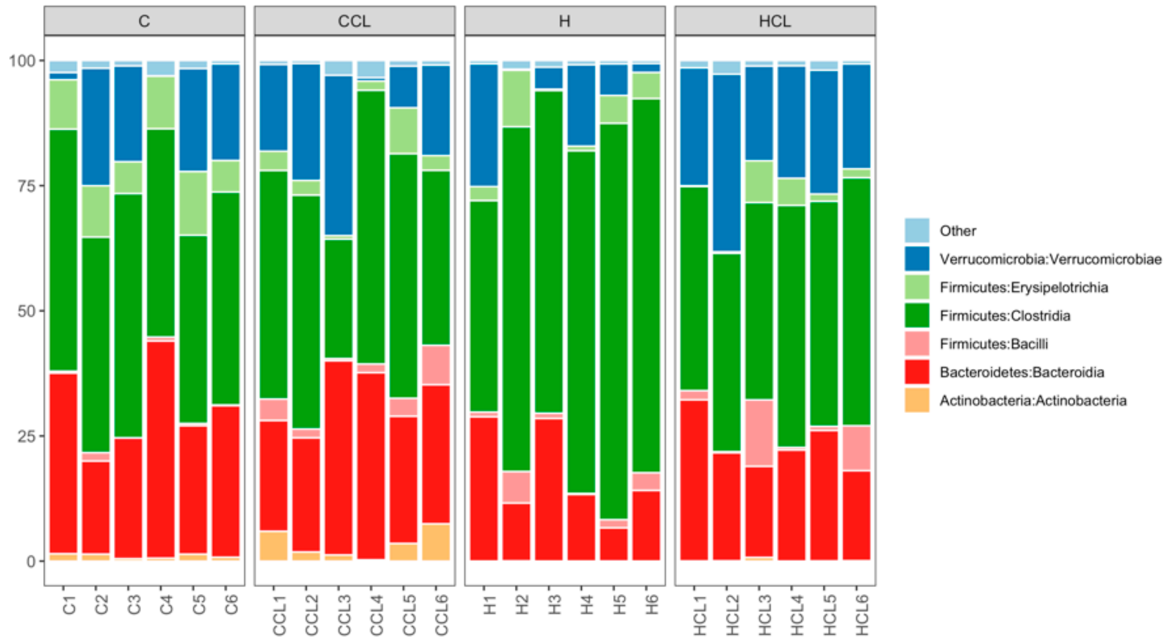

Figure S2. Taxonomic profiles of bacterial communities shown at the class level of all faecal samples. C, corn starch diet-fed rats; CCL, corn starch diet-fed rats supplemented with *Caulerpa lentillifera*; H, high-carbohydrate, high-fat diet-fed rats; and HCL, high-carbohydrate, high-fat diet-fed rats supplemented with *Caulerpa lentillifera*.

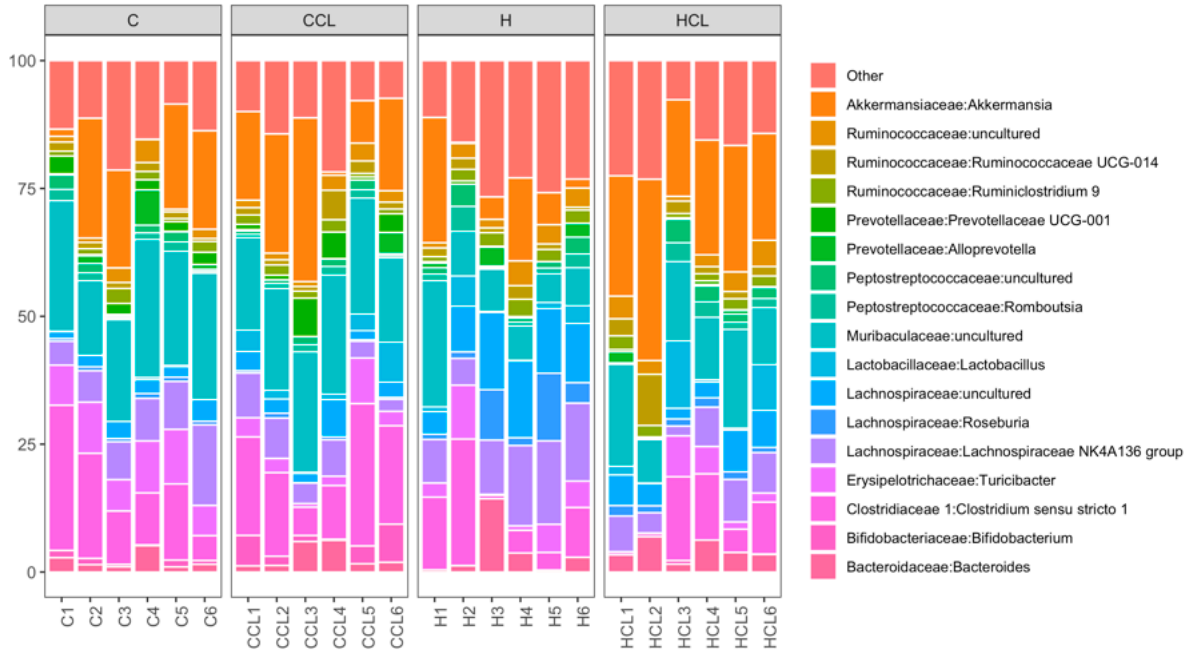

Figure S3. Taxonomic profiles of bacterial communities shown at the genus level of all faecal samples. C, corn starch diet-fed rats; CCL, corn starch diet-fed rats supplemented with *Caulerpa lentillifera*; H, high-carbohydrate, high-fat diet-fed rats; HCL, high-carbohydrate, high-fat diet-fed rats supplemented with *Caulerpa lentillifera*.

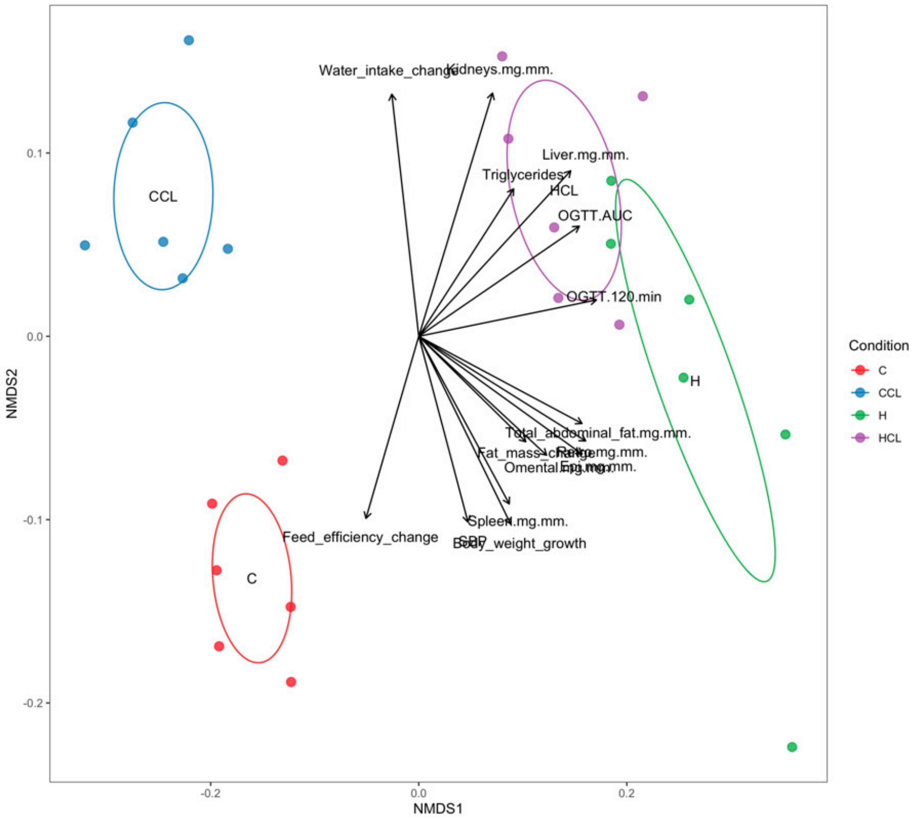

Figure S4. Correlation between bacterial community structure (points) and environmental variables (arrows). C, corn starch diet-fed rats; CCL, corn starch diet-fed rats supplemented with *Caulerpa lentillifera*; H, high-carbohydrate, high-fat diet-fed rats; HCL, high-carbohydrate, high-fat diet-fed rats supplemented with *Caulerpa lentillifera*.

Supplementary Tables

Table S1. PERMANOVAs based on Bray-Curtis similarity measure for square-root transformed abundances of all rat faecal samples.

Source	df	SS	MS	Pseudo-F	*p*(perm)	Unique Perms
Diet	1	9115.5	9115.5	8.8418	0.0001	9890
Treatment	1	2845.8	2845.8	2.7603	0.0001	9860
Diet × treatment	1	2185.7	2185.7	2.1201	0.0001	9849
Res	20	20619	1031			
Total	23	34766				
**PAIR-WISE TESTS**						
**Groups**				***t***	***p*(perm)**	**Unique Perms**
C, CCL				1.7178	0.0028	462
C, H				2.4170	0.0026	462
C, HCL				2.2273	0.0021	462
CCL, H				2.5635	0.0013	462
CCL, HCL				2.2596	0.0024	462
H, HCL				1.3972	0.0095	461

p-values were calculated using 9,999 permutations under a residual model. C, corn starch diet-fed rats; CCL, corn starch diet-fed rats supplemented with *Caulerpa lentillifera*; H, high-carbohydrate, high-fat diet-fed rats; HCL, high-carbohydrate, high-fat diet-fed rats supplemented with *Caulerpa lentillifera*.

Table S2. Correlation between bacterial community structure and physiological parameters ($p < 0.05$).

**Physiological Variables**	**R^2**	***p* value**
Oral glucose tolerance test—120-min blood concentration of glucose	0.76	0.001
Liver wet weight	0.76	0.001
Retroperitoneal fat	0.74	0.001
Epididymal fat	0.74	0.001
Oral glucose tolerance area under the curve	0.70	0.001
Total abdominal fat	0.69	0.001
Kidneys wet weight	0.58	0.001
Omental fat	0.50	0.002
Body weight	0.47	0.002
Water intake	0.46	0.002
Spleen wet weight	0.41	0.003
Plasma triglycerides	0.38	0.003
Fat mass	0.36	0.005
Systolic blood pressure	0.32	0.022
Feed efficiency	0.32	0.019

Table S3. Relative abundance of zOTUs affected by diet (ANOVA with *p* adjusted <0.05) between C, CCL, H and HCL rats.

OTU_ID	C (%)	CCL (%)	H (%)	CL%)	Phylum	Family	Genus
Zotu59	0.29	0.52	0.00	0.01	Actinobacteria	Bifidobacteriaceae	*Bifidobacterium*
Zotu80	0.11	0.58	0.00	0.00	Bacteroidetes	Muribaculaceae	unclassified
Zotu109	0.01	0.01	0.25	0.16	Bacteroidetes	Muribaculaceae	unclassified
Zotu608	0.00	0.00	0.01	0.02	Bacteroidetes	Muribaculaceae	unclassified
Zotu1036	0.05	0.03	0.00	0.00	Bacteroidetes	Muribaculaceae	unclassified
Zotu7	2.07	3.02	0.28	0.34	Bacteroidetes	Prevotellaceae	*Prevotellaceae UCG-001*
Zotu6	4.06	3.70	0.07	0.03	Firmicutes	Clostridiaceae 1	*Clostridium sensu stricto 1*
Zotu540	0.00	0.00	0.02	0.04	Firmicutes	Lachnospiraceae	*Acetatifactor*
Zotu212	0.08	0.15	0.00	0.00	Firmicutes	Lachnospiraceae	*Acetitomaculum*
Zotu352	0.01	0.00	0.07	0.03	Firmicutes	Lachnospiraceae	*Blautia*
Zotu510	0.00	0.00	0.03	0.04	Firmicutes	Lachnospiraceae	*Eisenbergiella*
Zotu26	1.16	0.95	0.00	0.00	Firmicutes	Lachnospiraceae	*Eubacterium ventriosum group*
Zotu55	0.01	0.01	0.82	0.37	Firmicutes	Lachnospiraceae	*Lachnospiraceae NK4A136 group*
Zotu124	0.00	0.00	0.14	0.36	Firmicutes	Lachnospiraceae	*Lachnospiraceae NK4A136 group*
Zotu169	0.08	0.26	0.00	0.00	Firmicutes	Lachnospiraceae	*Lachnospiraceae NK4A136 group*
Zotu208	0.00	0.00	0.20	0.08	Firmicutes	Lachnospiraceae	*Lachnospiraceae NK4A136 group*
Zotu217	0.00	0.00	0.02	0.22	Firmicutes	Lachnospiraceae	*Lachnospiraceae NK4A136 group*
Zotu224	0.01	0.00	0.19	0.07	Firmicutes	Lachnospiraceae	*Lachnospiraceae NK4A136 group*
Zotu307	0.00	0.00	0.19	0.04	Firmicutes	Lachnospiraceae	*Lachnospiraceae NK4A136 group*
Zotu391	0.01	0.10	0.00	0.00	Firmicutes	Lachnospiraceae	*Lachnospiraceae NK4A136 group*
Zotu790	0.00	0.00	0.02	0.01	Firmicutes	Lachnospiraceae	*Lachnospiraceae NK4A136 group*
Zotu374	0.00	0.00	0.06	0.07	Firmicutes	Lachnospiraceae	*Lachnospiraceae UCG-001*
Zotu28	0.00	0.00	1.66	0.74	Firmicutes	Lachnospiraceae	*Lachnospiraceae UCG-006*
Zotu204	0.00	0.00	0.29	0.07	Firmicutes	Lachnospiraceae	*Roseburia*
Zotu248	0.00	0.00	0.17	0.09	Firmicutes	Lachnospiraceae	*Roseburia*
Zotu548	0.00	0.00	0.02	0.04	Firmicutes	Lachnospiraceae	*Roseburia*
Zotu36	0.00	0.00	1.16	0.27	Firmicutes	Lachnospiraceae	unclassified
Zotu131	0.02	0.00	0.24	0.10	Firmicutes	Lachnospiraceae	unclassified
Zotu144	0.00	0.00	0.29	0.06	Firmicutes	Lachnospiraceae	unclassified
Zotu286	0.00	0.00	0.13	0.05	Firmicutes	Lachnospiraceae	unclassified
Zotu302	0.00	0.00	0.12	0.05	Firmicutes	Lachnospiraceae	unclassified
Zotu306	0.02	0.01	0.09	0.05	Firmicutes	Lachnospiraceae	unclassified
Zotu354	0.00	0.01	0.10	0.03	Firmicutes	Lachnospiraceae	unclassified
Zotu450	0.00	0.00	0.04	0.03	Firmicutes	Lachnospiraceae	unclassified
Zotu529	0.00	0.00	0.05	0.02	Firmicutes	Lachnospiraceae	unclassified
Zotu592	0.00	0.00	0.02	0.04	Firmicutes	Lachnospiraceae	unclassified
Zotu945	0.00	0.00	0.01	0.02	Firmicutes	Lachnospiraceae	unclassified
Zotu1018	0.02	0.03	0.00	0.00	Firmicutes	Lachnospiraceae	unclassified

Zotu192	0.00	0.00	0.13	0.10	Firmicutes	Peptococcaceae	unclassified
Zotu288	0.00	0.01	0.05	0.10	Firmicutes	Peptococcaceae	unclassified
Zotu129	0.21	0.14	0.00	0.00	Firmicutes	Ruminococcaceae	*Eubacterium coprostanoligenes* group
Zotu53	0.00	0.00	0.37	0.53	Firmicutes	Ruminococcaceae	*Ruminiclostridium 6*
Zotu71	0.00	0.00	0.51	0.24	Firmicutes	Ruminococcaceae	*Ruminiclostridium 9*
Zotu158	0.00	0.00	0.17	0.11	Firmicutes	Ruminococcaceae	*Ruminiclostridium 9*
Zotu173	0.00	0.00	0.15	0.13	Firmicutes	Ruminococcaceae	*Ruminiclostridium 9*
Zotu181	0.01	0.00	0.15	0.10	Firmicutes	Ruminococcaceae	*Ruminiclostridium 9*
Zotu33	0.94	0.38	0.00	0.00	Firmicutes	Ruminococcaceae	*Ruminococcaceae NK4A214*
Zotu166	0.15	0.09	0.00	0.00	Firmicutes	Ruminococcaceae	*Ruminococcaceae UCG-010*
Zotu687	0.00	0.00	0.01	0.03	Firmicutes	Ruminococcaceae	*Ruminococcaceae UCG-013*
Zotu170	0.00	0.00	0.17	0.12	Firmicutes	Ruminococcaceae	*Ruminococcaceae UCG-014*
Zotu90	0.23	0.27	0.00	0.01	Firmicutes	Ruminococcaceae	*Ruminococcus 1*
Zotu63	0.03	0.01	0.53	0.34	Firmicutes	Ruminococcaceae	unclassified
Zotu64	0.02	0.01	0.46	0.30	Firmicutes	Ruminococcaceae	unclassified
Zotu65	0.52	0.25	0.00	0.00	Firmicutes	Ruminococcaceae	unclassified
Zotu200	0.00	0.00	0.06	0.12	Firmicutes	Ruminococcaceae	unclassified
Zotu315	0.00	0.00	0.06	0.07	Firmicutes	Ruminococcaceae	unclassified
Zotu358	0.01	0.01	0.06	0.07	Firmicutes	Ruminococcaceae	unclassified
Zotu272	0.00	0.00	0.08	0.05	Tenericutes	Mollicutes RF39	unclassified

Differential abundance analysis was performed using Mvabund. C, corn starch diet-fed rats; CCL, corn starch diet-fed rats supplemented with *Caulerpa lentillifera*; H, high-carbohydrate, high-fat diet-fed rats; HCL, high-carbohydrate, high-fat diet-fed rats supplemented with *Caulerpa lentillifera*.

Table S4. Relative abundance of zOTUs affected by treatment (ANOVA with *P* adjusted <0.05) between C, CCL, H and HCL rats.

OTU_ID	C (%)	CCL (%)	H (%)	HCL%)	Phylum	Family	Genus
Zotu21	0.26	1.79	0.09	0.35	Bacteroidetes	Muribaculaceae	unclassified
Zotu1204	0.00	0.02	0.00	0.01	Firmicutes	Lachnospiraceae	unclassified

Differential abundance analysis was performed using Mvabund. C, corn starch diet-fed rats; CCL, corn starch diet-fed rats supplemented with *Caulerpa lentillifera*; H, high-carbohydrate, high-fat diet-fed rats; HCL, high-carbohydrate, high-fat diet-fed rats supplemented with *Caulerpa lentillifera*.

Table S5. Taxonomic assignments of the zOTUs strongly correlated with physiological parameters.

OTU_ID	Phylum	Family	Genus	Correlation with physiological parameters
Zotu 59	Actinobacteria	Bifidobacteriaceae	*Bifidobacterium*	Epididymal fat (−), liver wet weight (−), oral glucose tolerance test 120-min concentration (−), oral glucose tolerance test area under the curve (−), retroperitoneal fat (−), total abdominal fat (−)
Zotu 109	Bacteroidetes	Muribaculaceae	unclassified	Epididymal fat (+), kidneys wet weight (+), liver wet weight (+),oral glucose tolerance test 120-minute concentration (+), omental fat (+), retroperitoneal fat (+), spleen wet weight (+), total abdominal fat (+)
Zotu 21	Bacteroidetes	Muribaculaceae	unclassified	Oral glucose tolerance test 120-min concentration (−)
Zotu 7	Bacteroidetes	Prevotellaceae	*Prevotellaceae UCG-001*	Liver wet weight (−), oral glucose tolerance test 120-min concentration (−), oral glucose tolerance test area under the curve (−), retroperitoneal fat (−), total abdominal fat (−)
Zotu 6	Firmicutes	Clostridiaceae 1	*Clostridium sensu stricto 1*	Epididymal fat (−), kidneys wet weight (−), liver wet weight (−), oral glucose tolerance test 120-minute concentration (−), oral glucose tolerance test area under the curve (−), retroperitoneal fat (−), total abdominal fat (−)
Zotu 540	Firmicutes	Lachnospiraceae	*Acetatifactor*	Feed efficiency (−), kidneys wet weight (+), liver wet weight (+)
Zotu 212	Firmicutes	Lachnospiraceae	*Acetitomaculum*	Oral glucose tolerance test 120-min concentration (-)
Zotu 352	Firmicutes	Lachnospiraceae	*Blautia*	Body weight (+), epididymal fat (+), oral glucose tolerance test 120-minute concentration (+), oral glucose tolerance test area under the curve (+), omental fat (+), retroperitoneal fat (+), right ventricle wet weight (+), systolic blood pressure (+), total abdominal fat (+)
Zotu 510	Firmicutes	Lachnospiraceae	*Eisenbergiella*	Epididymal fat (+), kidneys wet weight (+), liver wet weight (+), omental fat (+), total abdominal fat (+)
Zotu 124	Firmicutes	Lachnospiraceae	*Lachnospiraceae NK4A136 group*	Feed efficiency (−), food intake (−), kidneys wet weight (+), liver wet weight (+)
Zotu 169	Firmicutes	Lachnospiraceae	*Lachnospiraceae NK4A136 group*	Plasma alanine transaminase (+)
Zotu 208	Firmicutes	Lachnospiraceae	*Lachnospiraceae NK4A136 group*	Epididymal fat (+), oral glucose tolerance test 120-minute concentration (+), oral glucose tolerance test area under the curve (+), retroperitoneal fat (+), right ventricle wet weight (+), total abdominal fat (+)
Zotu 217	Firmicutes	Lachnospiraceae	*Lachnospiraceae NK4A136 group*	Feed efficiency (−), food intake (−)

Zotu 224	Firmicutes	Lachnospiraceae	*Lachnospiraceae NK4A136 group*	Epididymal fat (+), liver wet weight (+), oral glucose tolerance test 120-minute concentration (+), oral glucose tolerance test area under the curve (+), omental fat (+), retroperitoneal fat (+), right ventricle wet weight (+), total abdominal fat (+), plasma triglycerides (+)
Zotu 307	Firmicutes	Lachnospiraceae	*Lachnospiraceae NK4A136 group*	Body weight (+), epididymal fat (+), fat mass (+), omental fat (+), retroperitoneal fat (+), spleen wet weight (+), total abdominal fat (+)
Zotu 55	Firmicutes	Lachnospiraceae	*Lachnospiraceae NK4A136 group*	Epididymal fat (+), oral glucose tolerance test area under the curve (+), omental fat (+), retroperitoneal fat (+), total abdominal fat (+)
Zotu 790	Firmicutes	Lachnospiraceae	*Lachnospiraceae NK4A136 group*	Epididymal fat (+), liver wet weight (+), oral glucose tolerance test 120-minute concentration (+), oral glucose tolerance test area under the curve (+), omental fat (+), retroperitoneal fat (+), right ventricle wet weight (+), total abdominal fat (+)
Zotu 374	Firmicutes	Lachnospiraceae	*Lachnospiraceae UCG-001*	Kidneys wet weight (+)
Zotu 28	Firmicutes	Lachnospiraceae	*Lachnospiraceae UCG-006*	Body weight (+), epididymal fat (+), fat mass (+), plasma non-esterified fatty acids (+), omental fat (+), retroperitoneal fat (+), total abdominal fat (+)
Zotu 248	Firmicutes	Lachnospiraceae	*Roseburia*	Body weight (+), epididymal fat (+), fat mass (+), omental fat (+), spleen wet weight (+), total abdominal fat (+)
Zotu 548	Firmicutes	Lachnospiraceae	*Roseburia*	Feed efficiency (−), kidneys wet weight (+), liver wet weight (+)
Zotu 1018	Firmicutes	Lachnospiraceae	unclassified	Liver wet weight (−)
Zotu 131	Firmicutes	Lachnospiraceae	unclassified	Epididymal fat (+), oral glucose tolerance test 120-min concentration (+), oral glucose tolerance test area under the curve (+), omental fat (+), retroperitoneal fat (+), right ventricle wet weight (+), systolic blood pressure (+), total abdominal fat (+)
Zotu 144	Firmicutes	Lachnospiraceae	unclassified	Body weight (+), epididymal fat (+), fat mass (+), liver wet weight (+), left ventricle + septum wet weight (+), oral glucose tolerance test 120-min concentration (+), omental fat (+), retroperitoneal fat (+), systolic blood pressure (+), spleen wet weight (+), total abdominal fat (+)
Zotu 286	Firmicutes	Lachnospiraceae	unclassified	Epididymal fat (+), liver wet weight (+), oral glucose tolerance test 120-min concentration (+), oral glucose tolerance test area under the curve (+), omental fat (+), retroperitoneal fat (+), spleen wet weight (+), total abdominal fat (+), plasma triglycerides (+)
Zotu 302	Firmicutes	Lachnospiraceae	unclassified	Body weight (+), epididymal fat (+), liver wet weight (+), omental fat (+), retroperitoneal fat (+), total abdominal fat (+)
Zotu 306	Firmicutes	Lachnospiraceae	unclassified	Liver wet weight (+), oral glucose tolerance test 120-min concentration (+), retroperitoneal fat (+)
Zotu 354	Firmicutes	Lachnospiraceae	unclassified	Epididymal fat (+), oral glucose tolerance test 120-min concentration (+), retroperitoneal fat (+), total abdominal fat (+)
Zotu 36	Firmicutes	Lachnospiraceae	unclassified	Body weight (+), epididymal fat (+), fat mass (+), liver wet weight (+), left ventricle + septum wet weight (+), oral glucose tolerance test 120-min concentration (+), omental fat (+), retroperitoneal fat (+), systolic blood pressure (+), spleen wet weight (+), total abdominal fat (+)
Zotu 450	Firmicutes	Lachnospiraceae	unclassified	Oral glucose tolerance test area under the curve (+), right ventricle wet weight (+)
Zotu 529	Firmicutes	Lachnospiraceae	unclassified	Body weight (+), epididymal fat (+), kidneys wet weight (+), liver wet weight (+), oral glucose tolerance test 120-min concentration (+), oral glucose tolerance test area under the curve (+), omental fat (+), retroperitoneal fat (+), spleen wet weight (+), total abdominal fat (+)
Zotu 945	Firmicutes	Lachnospiraceae	unclassified	Spleen wet weight (+)
Zotu 192	Firmicutes	Peptococcaceae	unclassified	Epididymal fat (+), retroperitoneal fat (+), total abdominal fat (+)

Zotu 288	Firmicutes	Peptococcaceae	unclassified	Feed efficiency (-), kidneys wet weight (+), liver wet weight (+)
Zotu 129	Firmicutes	Ruminococcaceae	*Eubacterium coprostanoligenes group*	Liver wet weight (−)
Zotu 53	Firmicutes	Ruminococcaceae	*Ruminiclostridium 6*	Feed efficiency (-), kidneys wet weight (+), liver wet weight (+), oral glucose tolerance test 120-minute concentration (+), oral glucose tolerance test area under the curve (+)
Zotu 158	Firmicutes	Ruminococcaceae	*Ruminiclostridium 9*	Body weight (+), epididymal fat (+), liver wet weight (+), oral glucose tolerance test 120-minute concentration (+), omental fat (+), retroperitoneal fat (+), spleen wet weight (+), total abdominal fat (+)
Zotu 173	Firmicutes	Ruminococcaceae	*Ruminiclostridium 9*	Kidneys wet weight (+), liver wet weight (+), oral glucose tolerance test 120-minute concentration (+), oral glucose tolerance test area under the curve (+), plasma triglycerides (+)
Zotu 181	Firmicutes	Ruminococcaceae	*Ruminiclostridium 9*	Body weight (+), epididymal fat (+), liver wet weight (+), oral glucose tolerance test 120-min concentration (+), omental fat (+), retroperitoneal fat (+), spleen wet weight (+), total abdominal fat (+)
Zotu 71	Firmicutes	Ruminococcaceae	*Ruminiclostridium 9*	Epididymal fat (+), oral glucose tolerance test 120-min concentration (+), oral glucose tolerance test area under the curve (+), omental fat (+), retroperitoneal fat (+), spleen wet weight (+), total abdominal fat (+)
Zotu 33	Firmicutes	Ruminococcaceae	*Ruminococcaceae NK4A214 group*	Liver wet weight (-)
Zotu 687	Firmicutes	Ruminococcaceae	*Ruminococcaceae UCG-013*	Kidneys wet weight (+)
Zotu 170	Firmicutes	Ruminococcaceae	*Ruminococcaceae UCG-014*	Oral glucose tolerance test 120-min concentration (+), oral glucose tolerance test area under the curve (+), plasma triglycerides (+)
Zotu 90	Firmicutes	Ruminococcaceae	*Ruminococcus 1*	Epididymal fat (−), kidneys wet weight (−), liver wet weight (−), oral glucose tolerance test 120-minute concentration (−), oral glucose tolerance test area under the curve (−), omental fat (−), retroperitoneal fat (−), total abdominal fat (−), plasma triglycerides (−)
Zotu 315	Firmicutes	Ruminococcaceae	unclassified	Epididymal fat (+), kidneys wet weight (+), liver wet weight (+), omental fat (+), retroperitoneal fat (+), spleen wet weight (+), total abdominal fat (+)
Zotu 358	Firmicutes	Ruminococcaceae	unclassified	Epididymal fat (+), feed efficiency (-), kidneys wet weight (+), liver wet weight (+), oral glucose tolerance test 120-min concentration (+), retroperitoneal fat (+), total abdominal fat (+)
Zotu 63	Firmicutes	Ruminococcaceae	unclassified	Epididymal fat (+), liver wet weight (+), oral glucose tolerance test 120-min concentration (+), oral glucose tolerance test area under the curve (+), omental fat (+), retroperitoneal fat (+), total abdominal fat (+)
Zotu 64	Firmicutes	Ruminococcaceae	unclassified	Epididymal fat (+), kidneys wet weight (+), liver wet weight (+), oral glucose tolerance test 120-min concentration (+), oral glucose tolerance test area under the curve (+), omental fat (+), retroperitoneal fat (+), total abdominal fat (+)
Zotu 272	Tenericutes	unclassified	unclassified	Epididymal fat (+), liver wet weight (+), oral glucose tolerance test 120-minute concentration (+), oral glucose tolerance test area under the curve (+), retroperitoneal fat (+), total abdominal fat (+)

This table includes the physiological parameters that were found to be strongly correlated ($p < 0.05$) with the bacterial community and incorporates OTUs that interact with at least 1 of these parameters ($n = 49$). Plus sign (+) indicates positive correlations, while minus sign (−) indicates negative correlations.
